# Supplementary material for: 11% efficiency solid-state dye-sensitized solar cells with copper(II/I) hole transport materials
Source: Nat Commun. 2017 Jun 9;8:15390. doi: 10.1038/ncomms15390 (PMC5472710; doi:10.1038/ncomms15390)
Supplement: Supplementary Information — Supplementary Figures, Supplementary Table and Supplementary References [file ncomms15390-s1.pdf]

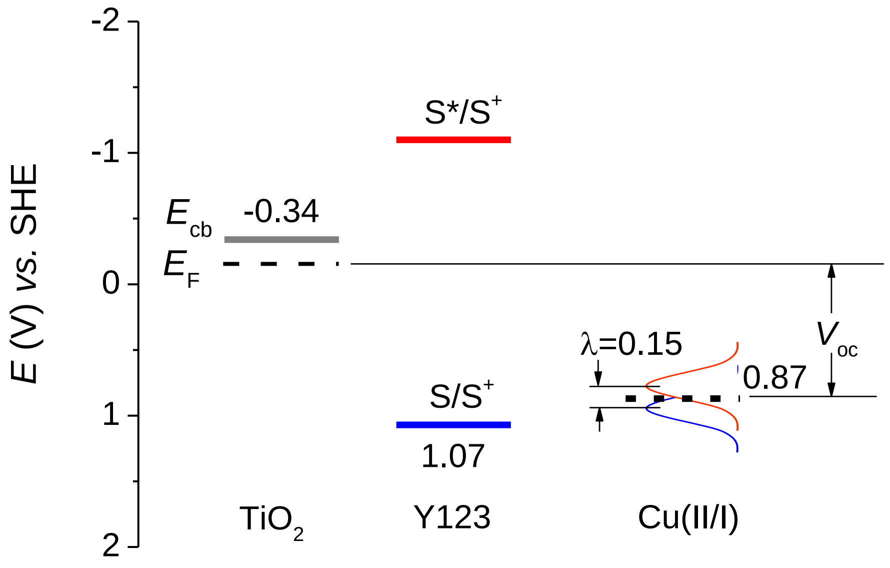

Supplementary Figure 1. A diagram of energy levels (*vs.* standard hydrogen electrode) of TiO_2, Y123 dye molecule, Cu(II/I) redox couple in the DSC. The V_{oc} is determined by the energy gap between the quasi-Fermi level (E_F) of TiO_2 and the redox potential of Cu(II/I). λ is the inner-sphere reorganization energy[1]. The blue and red curves are Gaussian distribution functions showing the density of occupied and unoccupied electronic states, respectively.

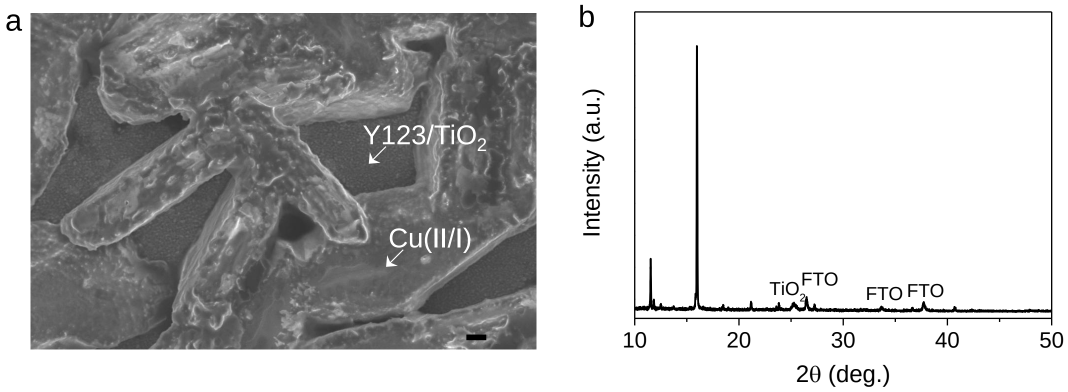

Supplementary Figure 2. (a) A SEM image of an electrode of FTO/TiO_2/Y123 having poor contact with the Cu(II/I) HTM. Scale bar: 2 μm. The unsealed DSC with the liquid electrolyte was kept under a lower pressure to accelerate the evaporation of solvents in the electrolyte. After drying, the PEDOT counter electrode was removed to obtain the sample FTO/TiO_2/Y123/HTM for SEM measurement. (b) XRD of the sample prepared in (a), showing reflection peaks from the HTM and indicated peaks from TiO_2 and FTO.

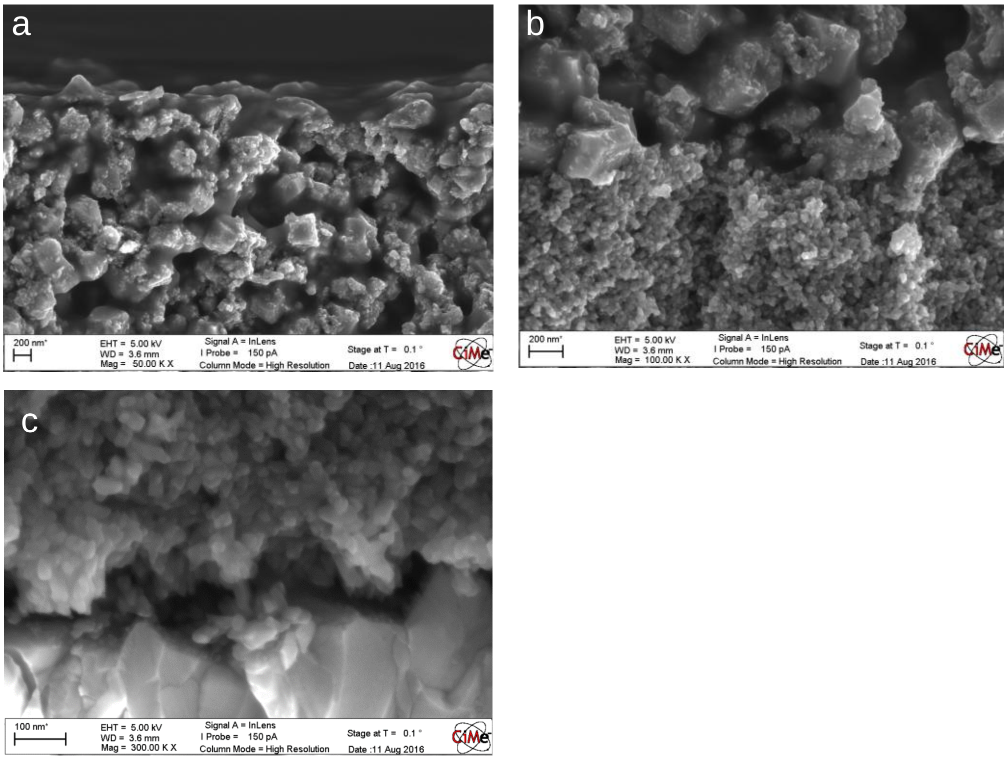

Supplementary Figure 3. Cross sectional SEM of a ssDSC based on Cu(II/I) HTM without the counter electrode. (a) The TiO_2 scattering layer alone. (b) The TiO_2 scattering layer coupled with the transparent TiO_2 layer. (c) The transparent TiO_2 layer alone on the FTO glass substrate.

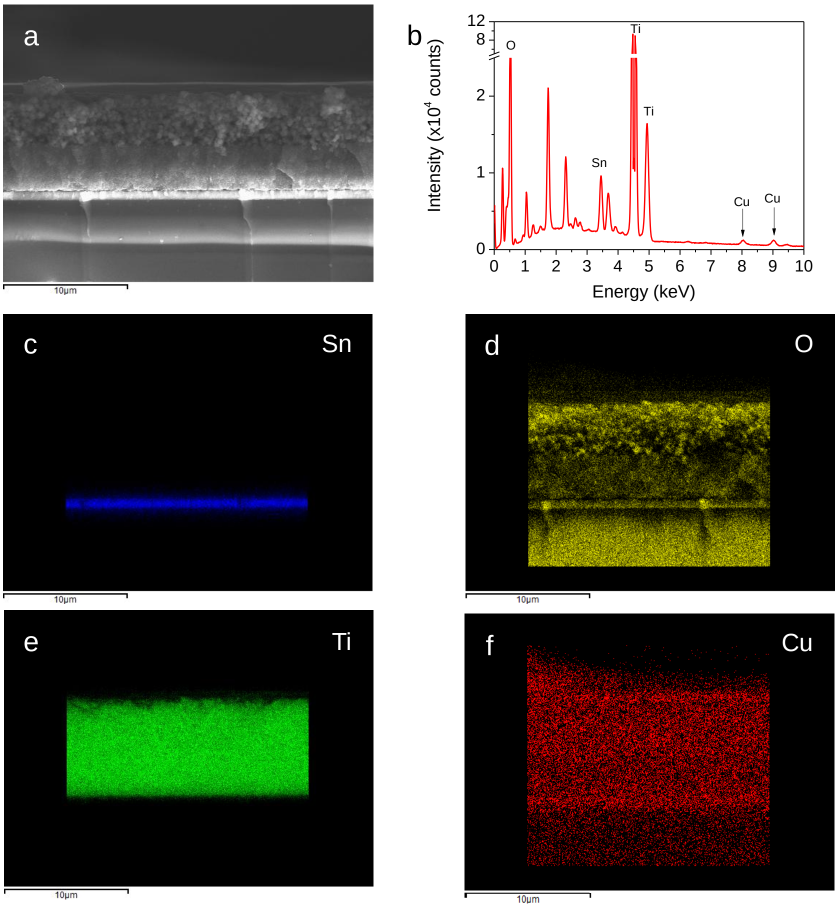

Supplementary Figure 4. (a) Cross sectional image of the Y123-sensitized TiO_2 film infiltrated with the Cu(II/I) HTM. (b) Energy dispersive spectroscopy indicating various elements. Elementary mapping of (c) Sn, (d) O, (e) Ti and (f) Cu.

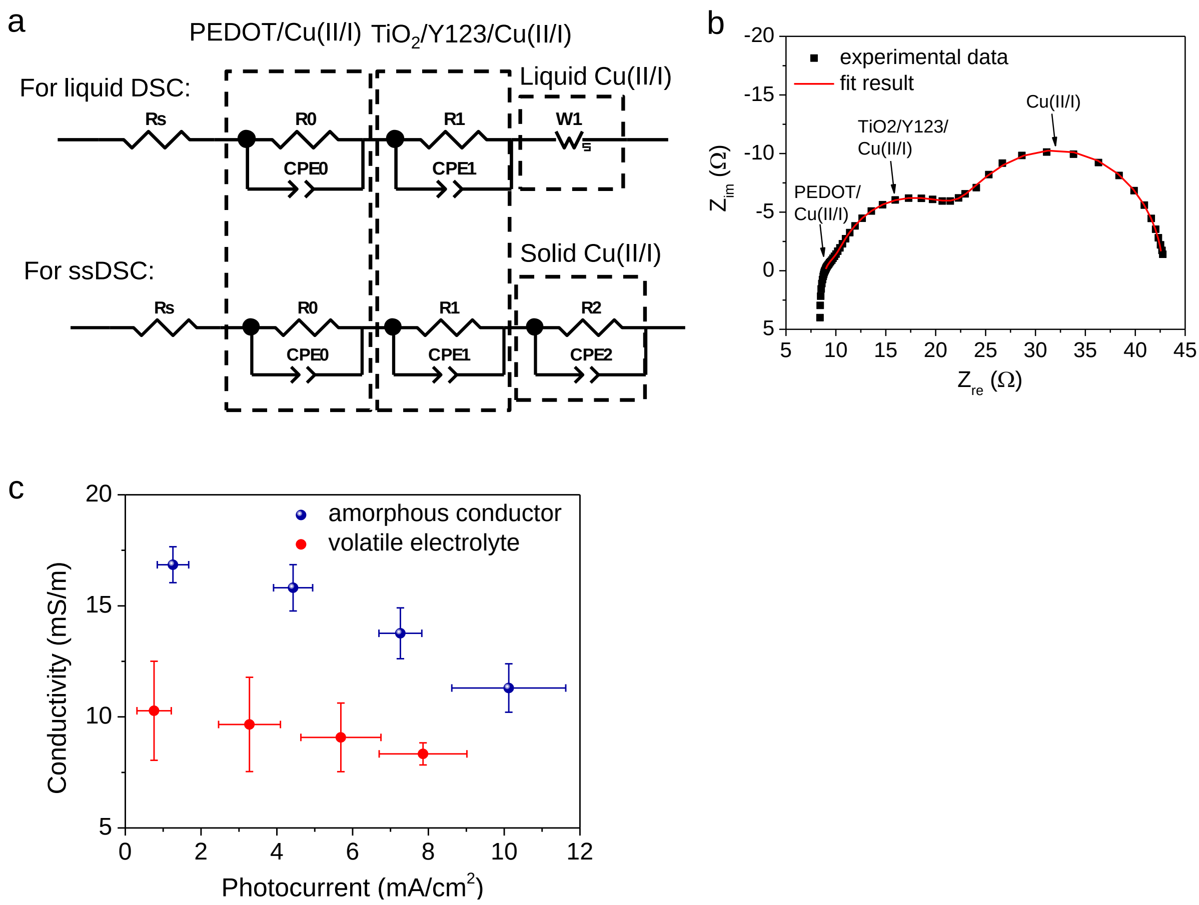

Supplementary Figure 5. (a) Equivalent circuit model to analyze EIS of DSCs based on the volatile electrolyte and the amorphous conductor. At intermediate and higher forward biases, electron transport resistance in TiO_2 is much lower than recombination resistance (R_{rec}). A simplified equivalent circuit[2], where the transmission line element is replaced by a parallel $R_{rec}C_{\mu}$ circuit, is used to fit the impedance spectra. For liquid DSCs, the equivalent circuit model is composed of a parallel $R_{rec}C_{\mu}$ in series with an RC element for the counter electrode and a Warburg diffusion resistance for the ionic transport in the electrolyte. For ssDSCs, we used a similar equivalent circuit model, where a $R_{hole}C_{hole}$ element replaces the Warburg impedance. (b) A typical spectroscopy of a DSC measured by EIS. The data can be best fitted by using the equivalent circuit models in (a). The arrow indicates frequency-dependent semi-circle (from high to low frequency) corresponding to the interface of PEDOT/Cu(II/I), the interface of TiO_2/Y123/Cu(II/I), and the Cu(II/I) transport material in DSCs. (c)

Photocurrent-dependent conductivity of the amorphous transport and the volatile electrolyte. The solar cells were measured under radiation at 1,000 W/m^2 provided by a white light-emitting diode (LED). The conductivity was calculated by $L/(A \times R)$, where L (around 9 μm) is the thickness of the hole conductor or electrolyte between the two FTO glasses, A (0.286 cm^2) is the photoactive area of DSCs and R is resistance obtained by EIS measurements. The average (symbols) and standard deviation (error bars) were calculated from solar cell numbering between four and six.

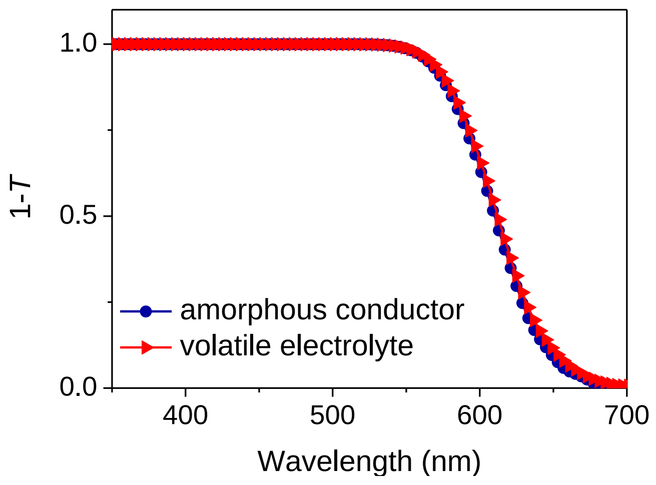

Supplementary Figure 6. (1-*T*) of dummy cells based on the amorphous Cu(II/I) HTM and the volatile electrolyte. *T* is transmittance of dummy cells. The structure of dummy cells is glass/TiO_2/Y123/HTM/glass. The thickness of transparent TiO_2 film is 3.5 μm.

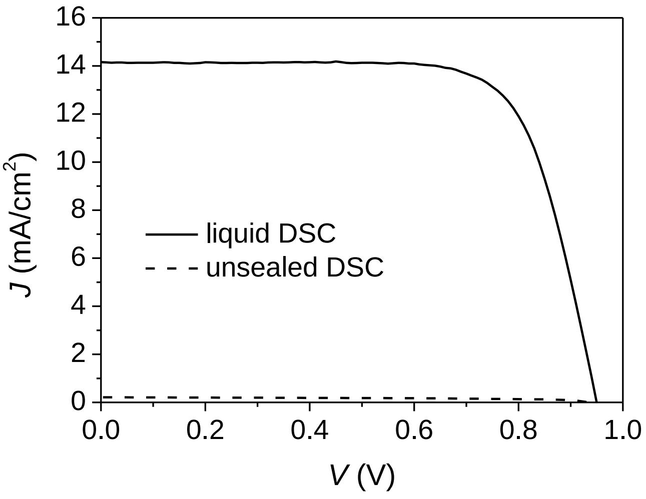

Supplementary Figure 7. *J*-*V* curves of a DSC with a *tris*-(bipyridine) cobalt(II/III) redox mediator electrolyte under AM1.5G conditions. The solid curve is *J*-*V* of the DSC with the liquid electrolyte (PCE = 9.8%, J_{sc} = 14.16 mA/cm^2, V_{oc} = 950 mV and FF = 0.732). The dash curve is *J*-*V* of the unsealed DSC by releasing solvents in the electrolyte for 150 min through an unsealed hole on a counter electrode (PCE = 0.1%, J_{sc} = 0.21 mA/cm^2, V_{oc} = 933 mV and FF = 0.552). The solar cell is composed of Y123-sensitized TiO_2 film (3.5 μm + 3 μm), a graphene based counter electrode[3], and a liquid electrolyte. The composition of electrolyte is 0.25 M $[Co(BPY)_3](BCN_4)_2$ (BPY = bipyridyl), 0.06 M $[Co(BPY)_3](BCN_4)_3$, 0.1 M LiTFSI and 0.6 M TBP in acetonitrile.

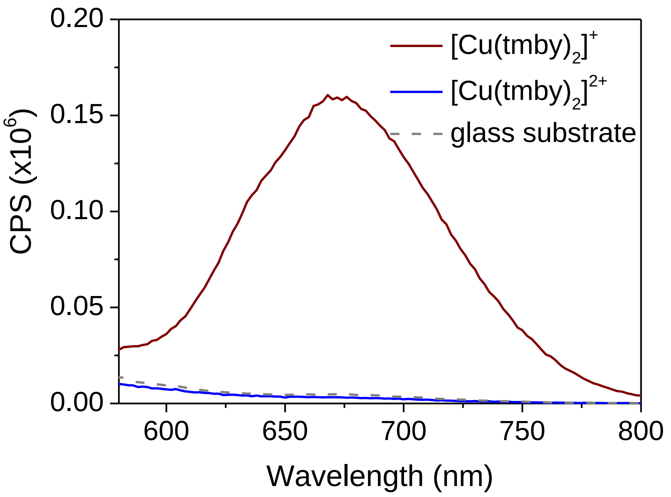

Supplementary Figure 8. Steady-sate photoluminescence spectra of $[Cu(tmby)_2](TFSI)$ and $[Cu(tmby)_2](TFSI)_2$ drop-casted on glass substrates from acetonitrile solution containing 0.1 M LiTFSI and 0.6 M TBP.

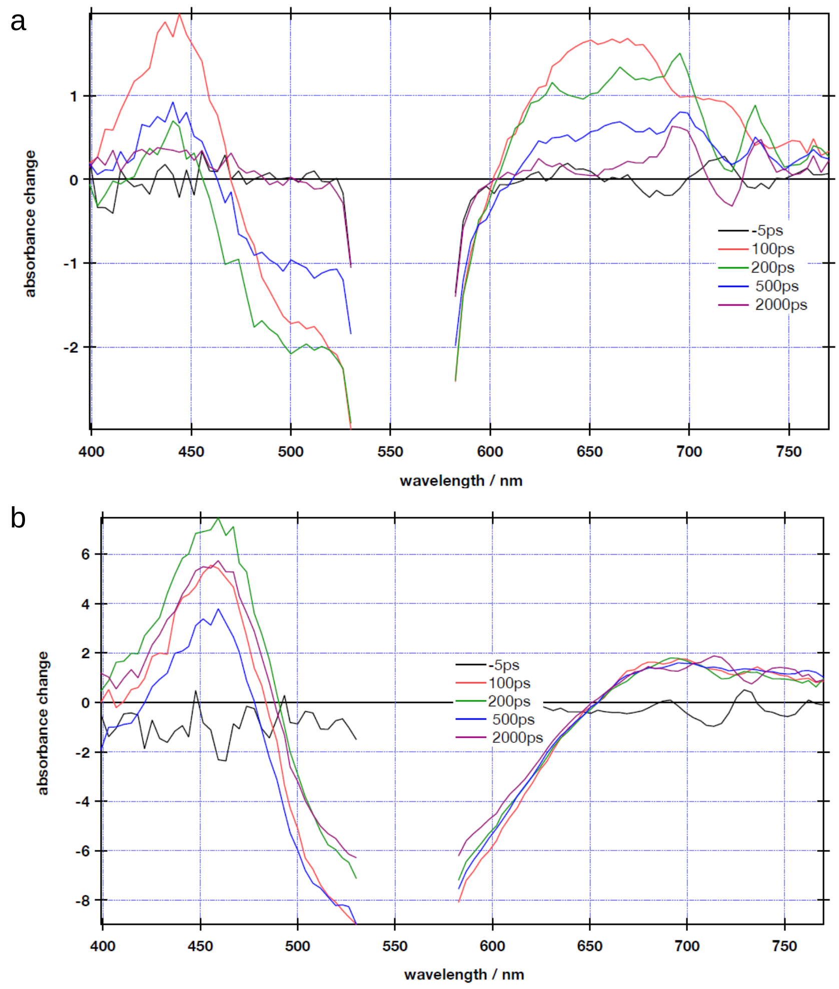

Supplementary Figure 9. Picosecond time resolved transient absorbance of (a) Al_2O_3/Y123/inert electrolyte and (b) TiO_2/Y123/inert electrolyte. The inert electrolyte contains 0.1 M LiTFSI and 0.6 M TBP in acetonitrile.

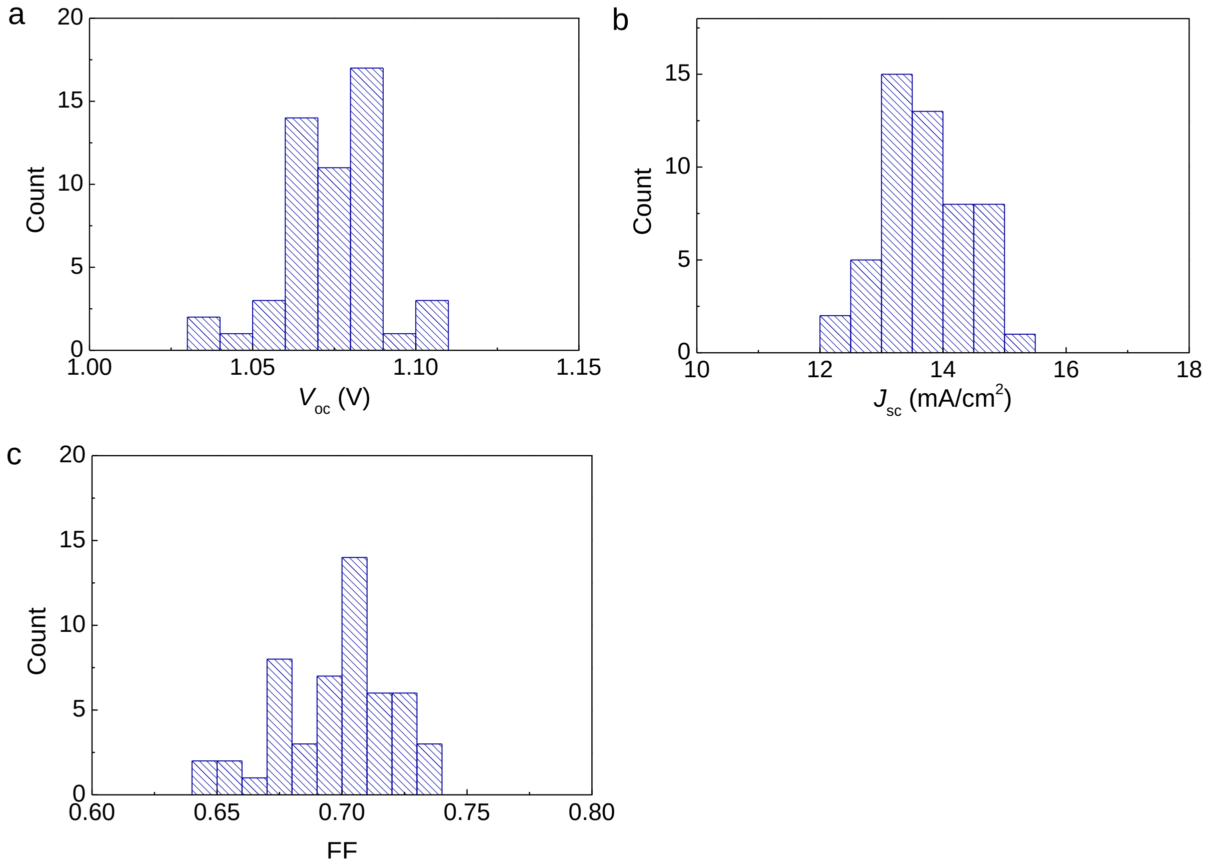

Supplementary Figure 10. Histogram of (a) V_{oc}, (b) J_{sc} and (c) FF of ssDSCs based on the Cu(II/I) HTM.

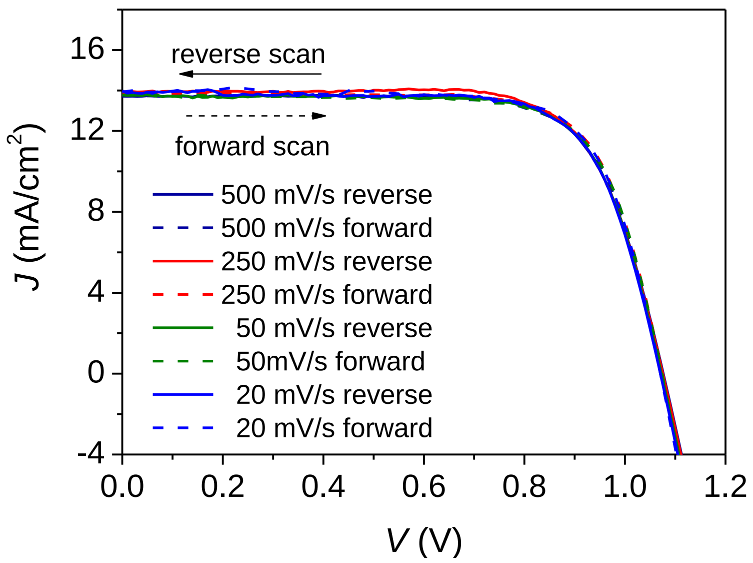

Supplementary Figure 11. Hysteresis test for an efficient ssDSC based on the Cu(II/I) HTM at different scanning rates.

Supplementary Table 1. Photovoltaic parameters of ssDSCs based on Cu(II/I) HTM with different thickness of mesoscopic TiO_2 films. The solar cells were measured under standard AM1.5G conditions.

TiO_2 film thickness (μm)	J_{sc} (mA/cm^2)	V_{oc} (V)	FF	PCE (%)
2.2[a]	10.21	1.09	0.72	8.0
3.5[a]	11.78	1.07	0.71	8.9
6.8[a]	11.40	1.05	0.64	7.7
3.5+3.0[b]	13.16	1.07	0.70	9.9

[a]The thickness of transparent mesoscopic TiO_2 films.

[b]The thickness of mesoscopic TiO_2 films composed of 3.5 μm-thick transparent mesoscopic TiO_2 layer + 3.0 μm-thick light scattering TiO_2 layer.

Supplementary references

1. Saygili, Y., Söderberg, M., Pellet, N., Giordano, F., Cao, Y., Munoz-Garcia, A. B., Zakeeruddin, S. M., Vlachopoulos, N., Pavone, M., Boschloo, G., Kavan, L., Moser, J.-E., Grätzel, M., Hagfeldt, A. & Freitag, M. Copper bipyridyl redox mediators for dye-sensitized solar cells with high photovoltage. *J. Am. Chem. Soc.* **138**, 15087–15096 (2016).
2. Boix, P. P., Larramona, G., Jacob, A., Delatouche, B., Mora-Seró, I. & Bisquert, J. Hole transport and recombination in all-solid Sb_2S_3-sensitized TiO_2 solar cells using CuSCN as hole transporter. *J. Phys. Chem. C* **116**, 1579-1587 (2012).
3. Mathew, S., Yella, A., Gao, P., Humphry-Baker, R., Curchod, B. F. E., Ashari-Astani, N., Tavernelli, I., Rothlisberger, U., Nazeeruddin, M. K. & Grätzel, M. Dye-sensitized solar cells with 13% efficiency achieved through the molecular engineering of porphyrin sensitizers. *Nat. Chem.* **6**, 242–247 (2014).
